# A simple blood biomarker based on gene expression describes cardiovascular health-related biological age

**DOI:** 10.1007/s11357-025-01784-6

**Published:** 2025-07-29

**Authors:** Natalia Hernández-Bellido, Adrián Hernández-Vicente, Laura García-Mendívil, Estel Ramos-Marquès, David Hernando, Alberto Cebollada, Ralf Köhler, Nuria Garatachea, Esther Pueyo, Laura Ordovás

**Affiliations:** 1 Biomedical Signal Interpretation and Computational Simulation (BSICoS) Group, Instituto de Investigación en Ingeniería de Aragón (I3A), Universidad de Zaragoza, Zaragoza, Spain; 2 BSICoS Group, Instituto de Investigación Sanitaria (IIS) Aragón, Zaragoza, Spain; 3 Growth, Exercise, Nutrition and Development (GENUD) Group, University of Zaragoza, Zaragoza, Spain; 4 Department of Physiatry and Nursing, Faculty of Health and Sport Sciences (FCSD), University of Zaragoza, Huesca, Spain; 5 Instituto Aragonés de Ciencias de La Salud (IACS), Zaragoza, Spain; 6 Fundación Agencia Aragonesa Para La Investigación y El Desarrollo (ARAID), Zaragoza, Spain; 7 Centro de Investigación Biomédica en Red de Fisiopatología de La Obesidad y Nutrición (CIBER-OBN), Madrid, Spain; 8 Instituto Agroalimentario de Aragón (IA2), CITA-Universidad de Zaragoza, Zaragoza, Spain; 9 Centro de Investigación Biomédica en Red de Bioingeniería, Biomateriales y Nanomedicina (CIBER-BBN), Zaragoza, Spain

**Keywords:** Biological age, Biomarker of aging, Physical activity, Autonomic nervous system modulation, Exercise intervention

## Abstract

The need to monitor the aging process as a risk factor for disease and mortality beyond chronological age (CA) has led to numerous investigations into the estimation of the biological age (BA) of individuals. However, the accuracy of BA estimation tools is often judged by their ability to approximate CA, questioning their value in capturing the variance in health status and thus correctly estimating BA. Their biological relevance is often assessed in relation to health outcomes or mortality, underexploiting their potential for real-time monitoring of BA. Furthermore, their complexity may limit their clinical translation to large populations. Here, we describe the gene expression-based age monitoring Clock (GamC), a simple biomarker of aging (BOA), and characterize its biological relevance with synchronous cardiovascular (CV) health-related functional data. GamC is calculated from the expression levels of three genes consistently dysregulated with age in blood (*ABLIM1*, *CCR7*, and *LEF1*). GamC shows moderate but reliable association with CA in three independent cohorts, supported by transcriptome-wide changes. It demonstrates specialized biological meaning, as it specifically describes current physical activity levels, but poorly correlates with autonomic nervous system function, both age-related factors associated with CV health. Finally, it expresses BA monitoring capacity by modestly responding to an effective exercise-based intervention in centenarians. In conclusion, GamC is proposed as a simple and affordable candidate BOA for reporting the individuals’ current CV health-related BA, thus promoting its broad translation and application into measures aimed at promoting healthy aging in relation to CV health, the leading cause of death worldwide.

**Supplementary Information:**

The online version contains supplementary material available at 10.1007/s11357-025-01784-6.

## Introduction

Aging, a major risk factor for disease, is an organismic process resulting from the progressive and cumulative decline in bodily function over time, which is built up from the molecular level. The molecular, cellular, and physiological changes that occur with aging are not uniform within and between individuals or populations. Therefore, aging is a much more complex biological process than just chronological age (CA). Biological age (BA), the age of the individual defined by the extent of age-dependent biological changes [1], captures the effect of both genetic and environmental factors acting over time. This effect is expressed in the individuals’ aging rate (AR), the difference between BA and CA. Fast and slow agers with early and late functional decline, respectively, translate into different disease susceptibilities and mortality risks [2]. Promoting healthy aging aims to achieve slow aging rates. Therefore, determining BA is crucial to gain insight into the role of specific mechanisms in the aging process, to predict the age-related disease/mortality risk of individuals and, given its environmentally modifiable nature [3, 4], to guide personalized preventive measures and potential therapeutic interventions that promote healthy aging, but, more importantly, to objectively evaluate their efficacy. This has led to a growing interest in the field of geroscience in describing biomarkers of aging (BOA) to estimate BA.

Many age prediction methods, clocks and predictors, have been reported to explore the association between age and age-related biological markers (reviewed extensively in [5]). However, the terms clock and predictor are used ambiguously and/or include mixed concepts that are difficult to separate. For example, their accuracy is often judged by their ability to approximate CA, being actually CA predictors, or their biological relevance is usually determined by their ability to predict mortality or disease, making them more appropriate as death/disease timers rather than current BA indicators [6]. In other cases, some methods even include CA as an age-related marker [2, 7, 8], in which case they are not strictly speaking aging clocks. Ideally, an aging clock should be an age-related index based on biological data, a BOA, related to an individual’s current age-related health status, their BA. The aging clock can also be a predictive biomarker of disease/mortality risk and, ideally, a response biomarker able to monitor changes in BA induced by healthy aging interventions. Instead, an age predictor attempts to accurately estimate CA using biological data, somehow losing its ability to capture the variance in health status of individuals and being more useful for other purposes such as forensics.

Different types of biological data have been used to estimate BA (reviewed in [1, 6]). Among these, BA indices based on gene and/or protein expression markers may be more sensitive to detect changes in the aging rates than, for example, clinical/physiological features, due to the rapidity of such changes and the sensitivity of the techniques used to quantify them [9]. Both protein and RNA-based indices have been successfully used to estimate BA [9–11]. Machine learning techniques and molecular omics data have also emerged as popular options for constructing age predictors and aging clocks, as they offer a wide range of applications even across different tissues, organs, and systems [6, 12]. However, they are highly complex and costly tools, which may limit their clinical translation. Furthermore, comparisons of BA estimators within the same [13–15] or independent study populations [1] have shown variability in the predictive power and biological information explained by each BA estimator, highlighting their complementary nature and the need for validation in independent populations. This is of concern because differences in age ranges, gender, ethnicity, sample size, and the data analysis strategy chosen can significantly affect the predictions and estimates of model performance [16, 17]. In any case, blood biomarkers offer significant advantages over current methods in terms of simplicity, cost-effectiveness, and scalability for clinical translation [18, 19].

Aging is one of the major risk factors for cardiovascular diseases (CVD), which are the leading cause of death worldwide. Thus, predicting cardiovascular (CV) health in relation to biological age could help to reduce the socioeconomic burden of CVD. Autonomic nervous system (ANS) regulation and physical activity (PA) levels are significant independent predictors of cardiovascular (CV) health and mortality [20, 21]. ANS regulation plays an important role during aging, leading to a gradual deterioration of body organs and functions, disrupting the homeostasis of the CV system and subsequently affecting the level of CV health of individuals [22]. Heart rate variability (HRV) is recognized as a robust method to assess the status of the ANS [23, 24] and is also an indicator of arrhythmic complications and a strong predictor of mortality and sudden death [25]. Indeed, there is a correlation of HRV markers with age, highlighting the intricate relationship between aging and the ANS [26]. In relation to PA, it is well known that PA declines with age, with older adults being the most physically inactive population group. PA levels are associated with the prevalence of risk factors that predispose individuals to the development of CV diseases [27] and with all-cause mortality [28]. Importantly, age modifies the relationship between PA and mortality, with its inverse association being stronger in the elderly [28]. In addition, Grässler et al. demonstrated the improvements in HRV markers, CV health, and CV risk factors in response to different levels of PA by using data from 26 studies [24], linking exercise and ANS modulation to CV health. Overall, the evidence suggests that indices reporting the ANS function and PA levels of individuals are relevant age-related phenotypes, so they are suitable for assessing the biological significance of novel BOAs in relation to CV health. Consequently, ANS- and/or PA-related BOAs can adequately monitor the current BA of individuals.

Here, we develop and characterize a novel and simple blood gene expression-based BOA, the Gene expression-based Age Monitoring Clock (GamC). GamC demonstrates an association with CA in three independent cohorts supported by whole transcriptome changes, thus confirming its age-related nature. Consistently, GamC associates with synchronous indicators of CV health of individuals (with differential sensitivity for variables that quantify the ANS activity and PA levels, which is indicative of the specific biological meaning of GamC) and shows a moderate sensitivity to an effective healthy aging intervention in centenarians, collectively demonstrating its biological relevance as an aging clock. Overall, this study proposes a simple and affordable BOA that gathers information about the current CV health status of individuals.

## Materials and methods

### Identification of consistently dysregulated age-related genes in blood

**Fig. 1:**
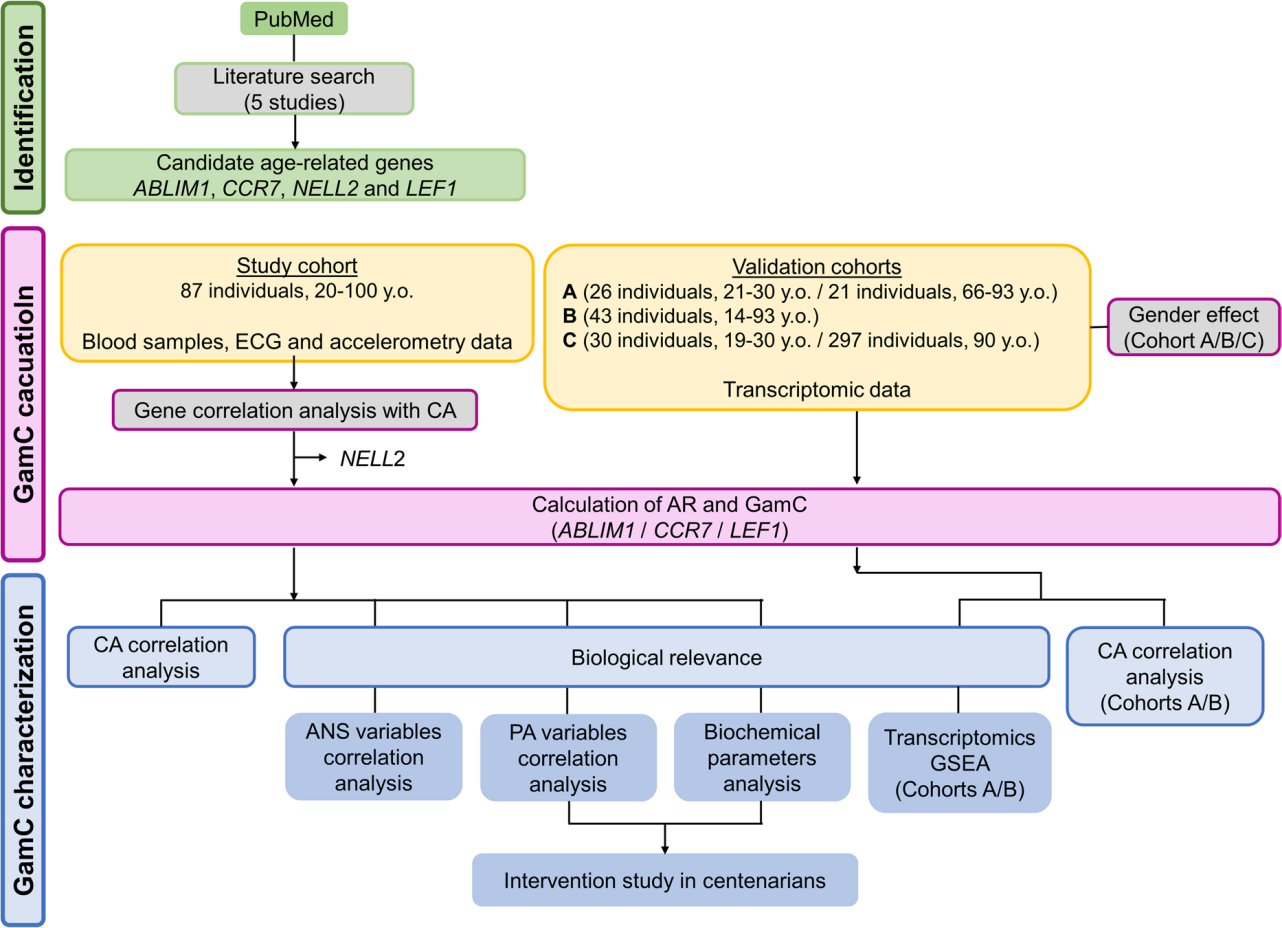
Workflow for the description and characterization of GamC. The study cohorts used for GamC calculation, validation and characterization are described in the yellow boxes, indicating the number of individuals, age range, and collected material or data. CA: chronological age, ECG: electrocardiogram, GEO: Gene Expression Omnibus; GSEA: Gene Set Enrichment Analysis, y.o.: years old

A literature search was conducted in PubMed to identify transcriptomic studies that have published lists of age-related differentially expressed genes in human blood. A cross-comparison of the lists resulted in the proposal of candidate genes for the construction of GamC (Fig. 1).

### Donors and samples of the study cohort

The study cohort is formed by a total of 87 Caucasian individuals from the region of Aragón (Spain) (Tables 1 and S1). The study was approved by the ethical committee for clinical research of Aragón (ID of the approval: PI17/0409 and PI18/381), and donors signed a written informed consent prior to enrollment. The study was conducted by adhering to the Declaration of Helsinki and in compliance with the European Union General Data Protection Regulation (EU 2016/679).

**Table 1:** Individuals in the study cohort: age range in years old (y.o.), total number of individuals, and percentage of males

Group	Age range (y.o.)	Number (% male)
Young	20–30	22 (100)
Adult	40–50	22 (100)
Elder	60–70	21 (100)
Centenarian	> 100	19 (26)

Subjects self-reported their date of birth and current medical conditions and medications. They were divided into four age groups (Table 1). Young, adult, and elderly individuals were excluded from the study if they suffered from acute illness, heart disease (such as heart failure or atrial fibrillation), were taking cardiac medication, or had any other clinical condition that contraindicated exercise. However, centenarians and donors who were overweight, sedentary, or had chronic diseases such as hypertension, diabetes, or hypercholesterolemia were included in the study, since they are representative of extreme longevity or have a high prevalence in society, respectively, and thus contribute to the power of GamC. Further demographic, clinical, and biometric data are shown in Table S1.

Whole blood samples, electrocardiogram (ECG) recordings, and PA data were synchronously collected from each individual along with their CA.

### Gene expression analysis by qPCR and generation of GamC

Whole blood samples were immediately processed to obtain peripheral blood mononuclear cells (PBMC) by using standard density gradient centrifugation procedures and stored at − 80 °C until use. RNA was extracted using the AllPrep DNA/RNA/miRNA Universal Kit (Quiagen). Next, 200 ng of RNA were reverse transcribed using the PrimeScript™ RT Reagent Kit (Takara). Quantitative PCR (qPCR) was performed using the NZYSupreme qPCR Green Master Mix Kit (Nzytech) with gene-specific oligonucleotide pairs (Table S2) in a Viia7 instrument (Thermo Fisher Scientific). The expression level of each age-related candidate gene was calculated as 2^−*ΔCt*^, where *ΔCt* = Ct_Gene_ − Ct_Reference_, being Ct_Gene_ the expression value of the gene and Ct_Reference_ the mean expression value of two reference genes, namely, *UBE2D2* and *GUSB2.*

The GamC of each individual was calculated as the sum of the AR and CA [29]. The AR was calculated according to previously described methods [30]. Briefly, the candidate genes with significant Pearson correlation coefficient (*R*) between their expression level and CA were selected to construct GamC (Fig. 1). The AR of each individual (*Δ*_*i*_) was obtained by linear regression of the expression levels (2^−*ΔCt*^) of each gene G against CA. The regression analysis returned the residual expression level of gene G in each individual i (*σ*_*G*,i_) and the regression factor associated with the gene G expression levels (*a*_*G*_). The *Δ*_*i*_ is calculated as the integration of all (*N*) gene-specific *Δ*_*G*,i_ (which is in turn obtained as the ratio between *σ*_*G*,i_ and *a*_*G*_) (Fig. 1) (Eq. (1)):

1 $${AR(\Delta }_{i})= \frac{1}{N} \sum_{G=1}^{N}{\Delta }_{G,i}= \frac{1}{N} \sum_{G=1}^{N}\frac{{\sigma }_{G,i}}{{a}_{G}}$$

### ECG data recording and HRV analysis

ECG recordings were obtained for all individuals in the study cohort (Fig. 1). A 12-lead high-resolution Holter ECG was acquired at rest with the 10 electrodes placed according to the manufacturer’s instructions (H12 +, Mortara Instrument, Milwaukee, WI, USA) and digitized at a sampling rate of 1000 Hz.

From the ECG recording, the heartbeats were detected and the RR interval time series were extracted using a multi-lead wavelet-based approach [31]. An operator manually verified each beat detection using a dedicated interface.

The 5-minute (min) RR interval series of each young, adult, and elderly subject was trimmed by removing 0.5 min from the beginning and the end, resulting in a final time series of 4 min. For the centenarian series, 4-min windows were defined within the 10-min recording in order to select the window with the lowest standard deviation (SD).

Linear and non-linear HRV indices were derived using algorithms developed and previously published in our research. These algorithms were implemented in MATLAB version R2021a (MATLAB, MathWorks Inc., Natick, MA, USA) [31–34]. The HRV markers extracted from the ECG are explained below:

#### Linear domain indices

##### Temporal domain indices

The time series of normal RR intervals after correction for ectopic beats [32] were denoted as NN and used for subsequent analyses. Several temporal HRV indices were derived from the NN interval series including mean heart rate (MHR) calculated as the inverse of the mean NN intervals, standard deviation of NN intervals (SDNN), standard deviation of the differences between adjacent NN intervals (SDSD), root mean square of consecutive differences of adjacent NN intervals (RMSSD), and percentage of consecutive NN intervals differing by more than 50 ms (ms) divided by the total number of all NN intervals (pNN50). SDNN is considered a long-term variability measure of total HRV power, representing the variability of the ANS activity. RMSSD, SDSD, and pNN50 are short-term variability measures of parasympathetic nervous system (PNS) activity.

##### Frequency domain indices

From the NN interval series, the instantaneous HR signal (dHR(*n*)) was derived using the integral pulse frequency modulation (IPFM) model, taking into account the presence of ectopic beats [32], and sampled at 4 Hz. This signal was high pass filtered (0.03 Hz) to remove the very low frequency components (dMHR(*n*)) and corrected as displayed in Eq. (2) [33]. This modulating signal ($m$($n$)) carries information about the ANS activity.

2 $$m\left(n\right)= \frac{\text{dHR }\left(n\right)-\text{dMHR }(n)}{\text{dMHR }(n)}$$

To estimate the spectral properties of the HRV signal, the Welch period programme was applied to *m*(*n*) using a Hamming window of 60 seconds (s) length with 30 s overlap. The power in the low frequency (LF) band, *P*_*LF*_, and the power in the high frequency (HF) band, *P*_*HF*_, were extracted by integrating the power in their respective bands: 0.04–0.15 Hz and 0.15–0.4 Hz, respectively. Normalized *P*_*LF*_ with the total power, named as *P*_*LFn*_, and the ratio *P*_*LF*_/*P*_*HF*_, as an indicator of the activity of both ANS branches, were also calculated.

#### Non-linear indices

Non-linear HRV indices, as non-stationary measures, were also derived from the RR interval series between the individual heartbeats. Detrended fluctuation analysis (DFA) was performed by extracting the correlations between successive RR intervals at different time scales in order to measure fluctuations at different scales to detect short- and long-term correlations (*α*_1_ and *α*_2_, respectively) [35]. These markers are used to assess the complexity and adaptability of the ANS.

Finally, SD1 and SD2 were extracted from the Poincaré plot by plotting each RR interval against the previous interval to create a scatter plot. SD1 and SD2 measure the short-term and long-term beat-to-beat variability of the RR interval series, respectively. The ratio SD1/SD2 was also calculated, as the ratio between the two variabilities in the RR time series [35]. These measures are used to investigate the structure and complexity of the interbeat intervals.

### Accelerometer data recording and analysis

Accelerometer data recordings were obtained for all individuals in the study cohort (Fig. 1). All individuals were asked to wear GENEActiv triaxial accelerometers (ActivInsights Ltd., Cambridgeshire, UK) for 24 h over seven consecutive days. These accelerometers were placed on the non-dominant wrist and programmed to record accelerations at 10 Hz, a frequency previously validated to classify daily activities (Zhang et al. 2012). Initialization of the GENEActiv accelerometers and retrieval of data in binary format were performed using GENEActiv PC (version 3.2) (ActivInsights Ltd., Cambridgeshire, UK).

The GGIR 3.0–2 package of the statistical programming language R v.4.3.2 [36] was used to perform accelerometer data analysis. Non-wear time detection and minimum valid time requirements for each accelerometer register were evaluated using GGIR default settings to facilitate comparability with previous studies. The minimum valid hours per day were set to sixteen, while the minimum valid days per record were set to two. Table S3 shows the relationship between the nomenclature used in this article and the variable names from the GGIR output.

Accelerometer variables were calculated [37], including the MX metrics, the average acceleration (AA), and the intensity gradient (IG). The MX metrics [38] assess the acceleration of the most active X min of a participant’s daily activity (e.g., M30 refers to the acceleration at which the most active 30 min were spent). These metrics can be used to describe the distribution of intensities over different time periods and allow for direct comparison with health-related PA guidelines. Intensity levels corresponding to the most active 1, 2, 5, 10, 15, 20, 30, 45, 60, 120, 240, 360, 480, 600, and 720 min were recorded. AA reflects the average acceleration throughout the entire measurement period and can be used as a proxy for total daily PA-related energy expenditure or PA volume [37, 39]. IG, calculated as the slope (negative) of the linear regression between the natural logarithm of time and acceleration intensity, captures the distribution of PA intensity.

Despite knowing the limitations of traditional cut-points [40, 41] due to their widespread use, the following indices were also calculated: Inactive time (IT: < 3 0 mg), time spent in light intensity PA (LPA: 30–10 0 mg), moderate intensity PA (MPA: > 100–400 mg), vigorous intensity PA (VPA: > 400 mg), and moderate-vigorous intensity PA (MVPA: > 100 mg) [42].

### Exercise intervention

For the exercise intervention (Fig. 1), centenarians were randomly assigned to the control or intervention group using a computer-generated shuffle list. Participants in the control group maintained their usual PA. The intervention group performed twice per week, non-consecutive, resistance training sessions over 12 weeks (24 sessions in total). All the sessions were performed one-on-one in the gym of the geriatric nursing home and were supervised by an experienced (5 years) strength and conditioning trainer (MSc in Sports Science). After the initial assessment, participants were enrolled in a resistance exercise routine consisting of 8 different exercises according to their Functional Ambulation Classification (FAC) level [43].

Each session lasted 40–60 min, including a 10-min warm-up and 30–50 min of resistance training. The warm-up consisted of one set of eight single-joint seated exercises without resistance, 10 repetitions, and 30-s rest between exercises. Resistance training consisted of 1–3 sets of the 8 exercises routine adapted to FAC (8–10 repetitions as fast as possible, at 50–70% of the one-repetition maximum) with resting periods of 1 min between exercises and 3–5 min between sets [44]. Load, number of sets, and type of exercise routine were adjusted to the new level of physical capacity every 2 weeks.

Whole blood samples and accelerometer variables were obtained at the end of the 3-month intervention for both control and intervention groups as described above. Biochemical analysis for total cholesterol, low-density lipoproteins (LDL), triglycerides, creatine kinase (CK) and glucose levels were conducted by Centro Inmunológico de la Comunidad Valenciana (CIALAB). The accelerometer variables were obtained as described before. For each PA variable, biochemical parameter and GamC, the change in the value of each parameter after the intervention period in each individual (*P*_*i*_) was calculated as the difference between after (II) and before (I) the intervention (*ΔP*_*i*_). Then, the median and interquartile range (IQR) of *ΔP*_*i*_ for each PA variable, biochemical parameter and GamC were calculated for the control and intervention groups.

### Validation cohorts and transcriptomic analysis

The literature search of point 2.1 and a search conducted in the GEO DataSets database, were filtered to extract studies containing readily accessible bulk tissue transcriptomic datasets from PBMC (as the study cohort) from young to elder individuals. Three independent transcriptomic datasets were obtained for analysis: cohort A (study from the USA, two age groups being 21–30 and > 70 y.o.) [45], cohort B (study from Spain, continuous ages from 19 to 93 y.o.) [46] and cohort C (study from Finland, two age groups being 19–30 and 90 y.o.) [47] (Fig. 1 and Table S4). Data were retrieved using the GEOquery package in R software, and gene annotation was applied to assign biological identifiers and filter out unannotated genes. Expression values were normalized and standardized by a quantile method for further analysis.

GamC was calculated as described above in cohorts A and B, but using the normalized and standardized expression values of the GamC-building genes. Cohort C was excluded from this calculation due to the lack of age variability in the older group.

Differential expression analysis (DEA) was performed in cohorts A and B using the Limma package in R software. Individuals were ranked by chronological age and GamC values, then divided into quartiles: young/first quartile (*Q*_1_) and elderly/fourth quartile (*Q*_4_) (Table S4). A linear model was fitted to the normalized and standardized expression data, including contrast matrices to define age-based comparisons. Log fold-change (logFC) values were estimated, and gene set enrichment analysis (GSEA) was performed using logFC values to identify enriched or depleted biological processes (GO terms) with a false discovery rate (FDR) ≤ 0.05. The top 50 GO terms with the highest absolute normalized enrichment score (NES) were manually classified into broader functional categories.

### Statistical analysis

Association analysis between two variables was performed using Pearson correlation analysis for the calculation of GamC [30], its association with CA, and the association of AR with CA. Spearman correlation analysis was used for the assessment of the association between independent HRV markers and PA variables with CA or GamC and also between HRV markers with PA variables.

Comparisons between unpaired observations in two independent groups were assessed using the Mann–Whitney *U* test.

As normality could not be assumed in the intervention study due to the small group sizes, the Wilcoxon matched-pairs signed rank test was performed in values of control and intervention groups independently to examine differences on *ΔP*_*i*_ of PA variables, biochemical parameters and GamC. The magnitude of the effect size (|r|) was calculated as described in Eq. (3), where *Z* represents the *Z*-score for the Wilcoxon matched-pairs signed rank test and *n* is the total number of observations [48].

3 $$|r|= \frac{Z}{\sqrt{n}}$$

The |r| was considered small if |r|= 0.1, medium if |r|= 0.3 and large if |r|= 0.5 [49].

All statistical analyses were performed using GraphPad Prism 8.0. The significance level was set at *P* ≤ 0.05 or, when adjusted for multiple-testing correction, at FDR ≤ 0.05, as conveniently indicated in the text and figure legends. The numerical value of P or FDR of every statistical test can be found in the supplementary tables.

## Results

### GamC is associated with CA

The calculation of the BOA started by identifying genes whose expression was consistently and significantly dysregulated with CA across independent study cohorts in PBMC or whole blood (Fig. 1). We performed a literature search seeking bulk tissue transcriptomic studies from different geographic origins (in some cases of different ancestries), hypothesizing that such genes, when combined in the BOA, would be able to account for genetic and environmental differences, including cultural and lifestyle, in relation to CA. A total of five independent studies were identified, which included variable geographic origins and age ranges from young to centenarians [11, 47, 50–52] (Table S5). A manual cross-comparison of the published lists of age-related differentially expressed genes (DEG) revealed four consistently dysregulated genes across all studies: *ABLIM1*, *CCR7*, *LEF1* and *NELL2*. These were selected as candidates for the construction of GamC.

**Fig. 2:**
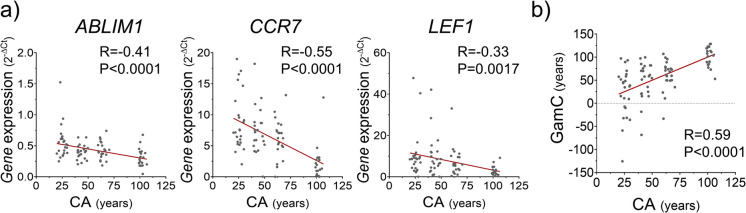
Evaluation of the relation with age of the candidate genes and GamC. **a** Pearson’s correlation analysis of the gene expression levels in PBMC (obtained by qPCR) of the age-related candidate genes and CA in the study cohort. **b** Pearson’s correlation analysis of GamC with CA. Pearson’s correlation coefficient (*R*), *P*-value (*P*), and linear regression (red line) are shown

Their potential to define a relevant BOA was evaluated in blood samples from a study cohort composed of 87 individuals with varying overall health and ages ranging from 20 to over 100 y.o. (Fig. 1 and Tables 1-S1). In agreement with the studies of the identification section of the workflow (Fig. 1 and Table S5) [11, 47, 50–52], *ABLIM1*, *CCR7*, and *LEF1* showed a significant negative correlation with CA in the study cohort (*R* = − 0.41, − 0.33, and − 0.55, respectively and all *P *≤ 0.05), confirming their association with age (Fig. 2a and Table S1). *NELL2* instead was not significantly correlated with CA (*R *= -0.18, *P *= 0.1) (Fig. S1) and therefore, it was excluded from the following steps of the study.

Next, *ABLIM1*, *CCR7*, and *LEF1* were used to construct the AR and GamC for each individual (Fig. 1). GamC was positively and significantly correlated with CA (*R* = 0.59). The degree of association exceeded that observed for the individual genes (Fig. 2b), demonstrating an improved explanatory power of this gene combination to describe CA. The AR was not significantly correlated with CA (Figure S2a), consistent with its expected individual-dependent nature.

These results indicate that GamC is associated with CA and, consequently, that the molecular data used to construct it contributes to describing this association.

### GamC association with age is maintained across independent study cohorts and at the transcriptome-wide level

The capacity to describe the age of GamC was analyzed in independent validation cohorts (Fig. 1). As the study cohort was mainly composed of male donors (Table S1), first, the influence of gender on this relationship was studied (Fig. 1). Using bulk PBMC transcriptomic data from three different cohorts of variable geographic origin (cohorts A – 34 % of males-, B - 51 % of males and C - 29 % of males) (Table S4) [45–47], a general downregulation of *ABLIM*, *CCR7*, and *LEF1* with CA was observed in both genders in each dataset, but also globally. The association was significant in all cases except for *ABLIM1* and *CCR7* in the segregated groups of cohort B, for which only a moderate non-significant correlation was observed (Figure S3 and Table S4). Thus, *ABLIM*, *CCR7*, and *LEF1* were generally associated with CA in a cohort- and gender-independent manner. Consequently, GamC was calculated without gender segregation for cohorts A and B (Fig. 1). Calculation of GamC for cohort C was not mathematically possible and was therefore excluded from further analysis.

**Fig. 3:**
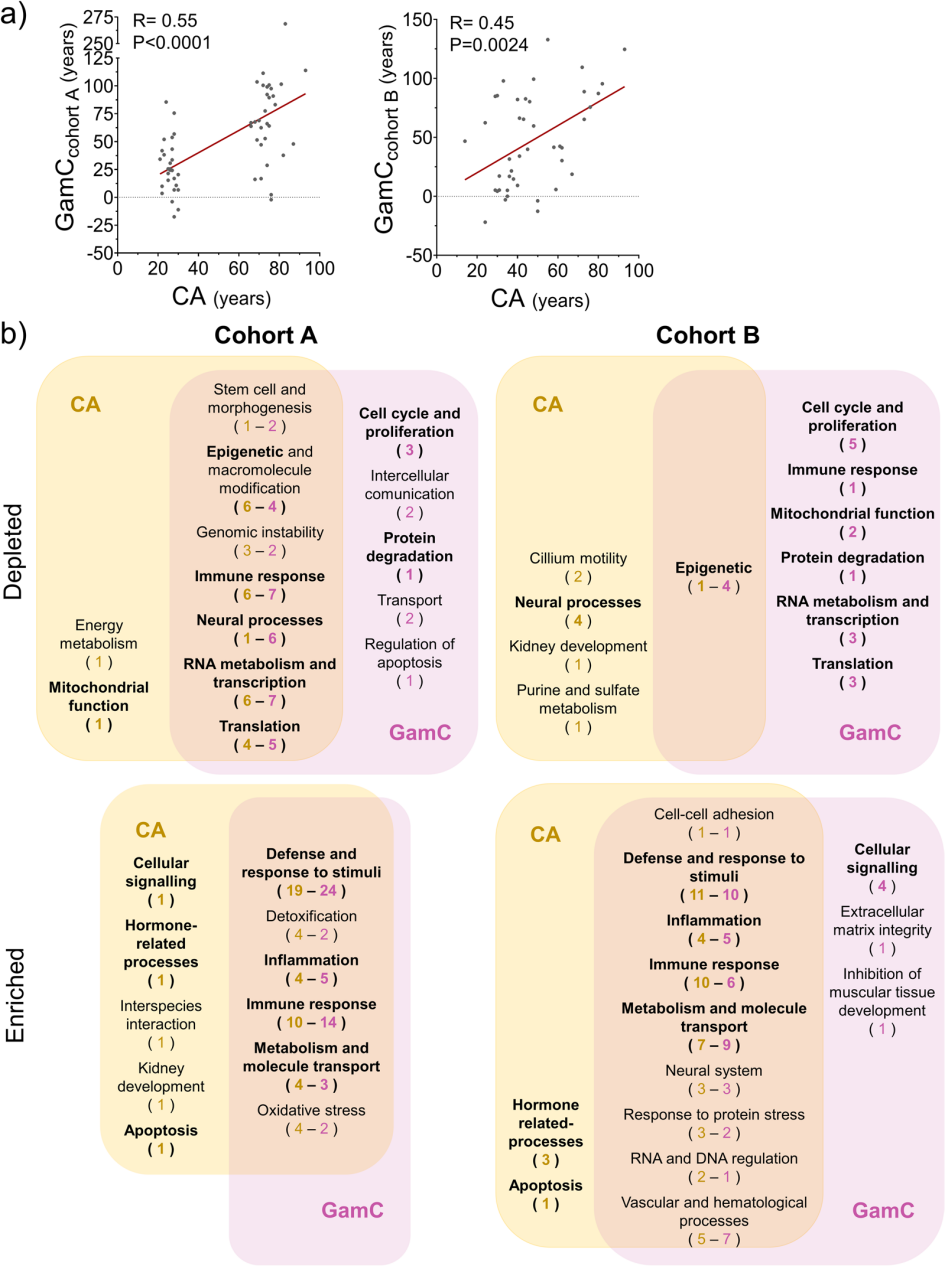
Validation of GamC in independent cohorts and evaluation of its biological relevance at the molecular level. **a** Evaluation of the association between GamC (constructed from transcriptomic data) and CA in the validation cohorts A (left) and B (right). Pearson’s correlation coefficient (*R*), *P*-value (*P*), and linear regression (red line) are shown. **b** Gene set enrichment analysis in the transcriptomic datasets of the validation cohorts A and B. Enriched and depleted functional categories in the elderly classified by CA or GamC are shown (bold letters are used for categories common between CA and GamC and/or cohorts A and B). The number of gene ontologies belonging to each functional category is indicated in brackets and color coded (CA in yellow or GamC in pink)

Consistent with the results observed in the study cohort, GamC maintained a significant positive correlation with CA in the validation cohorts A and B with *R* = 0.55 and 0.45, respectively (Fig. 3a) that was independent of the AR (Figure S2b-c and Tables S4−S6), demonstrating its age-related relevance across populations.

Then, the biological significance of GamC was examined by analyzing its ability to capture age-related transcriptomic changes (Fig. 1). GSEA revealed that, in general, the degree of overlap of functional processes dysregulated with CA and GamC between cohorts was high. Only the depleted processes of cohort B seemed differential, but those of GamC mostly matched the depleted ones of cohort A, suggesting that GamC in cohort B may better describe the age-related changes in gene expression than CA itself (Fig. 3b and Table S7). Overall, both cohorts retrieved processes previously reported to be altered with aging (Table S4) [53] and/or found dysregulated in the studies of the identification section of the workflow (Table S5). Enriched processes included, for example, defense and response to stimuli, immune response, inflammation or metabolism and molecule transport, whereas depleted processes related to mitochondrial function, RNA metabolism and transcription, translation and proteostasis or epigenetics. Therefore, GamC is able to capture the transcriptomic changes that occur with CA, confirming its age-related nature in different cohorts also at the molecular level.

Thus, the relationship between GamC and age is robust across independent cohorts of different geographic origin and is supported by a molecular basis. Of note, GamC can also be calculated from different sources of gene expression data, namely qPCR and transcriptomic.

### GamC marginally describes age-related changes in the ANS function

Once the relationship between GamC and CA was established, we studied the biological relevance of GamC. For this, we observed the ability to predict the current CV health of individuals in terms of ANS function and PA levels (Fig. 1). Because of the established relationship between ANS and PA with age and, in turn, in defining CV health, we analyzed the general relationship between HRV and PA markers hypothesizing that those correlated would be stronger age-related functional indicators. Accelerometer and HRV markers were only partially significantly associated (116 out of 308 tested correlations had FDR ≤ 0.05). In particular, some frequency domain (*P*_*LFn*_, *P*_*LF*_/*P*_*HF*_) and non-linear domain indices (α_1_, α_2_ or SD1 and SD2) had no relationship with virtually any of the PA markers (Figure S4 and Table S9).

**Fig. 4:**
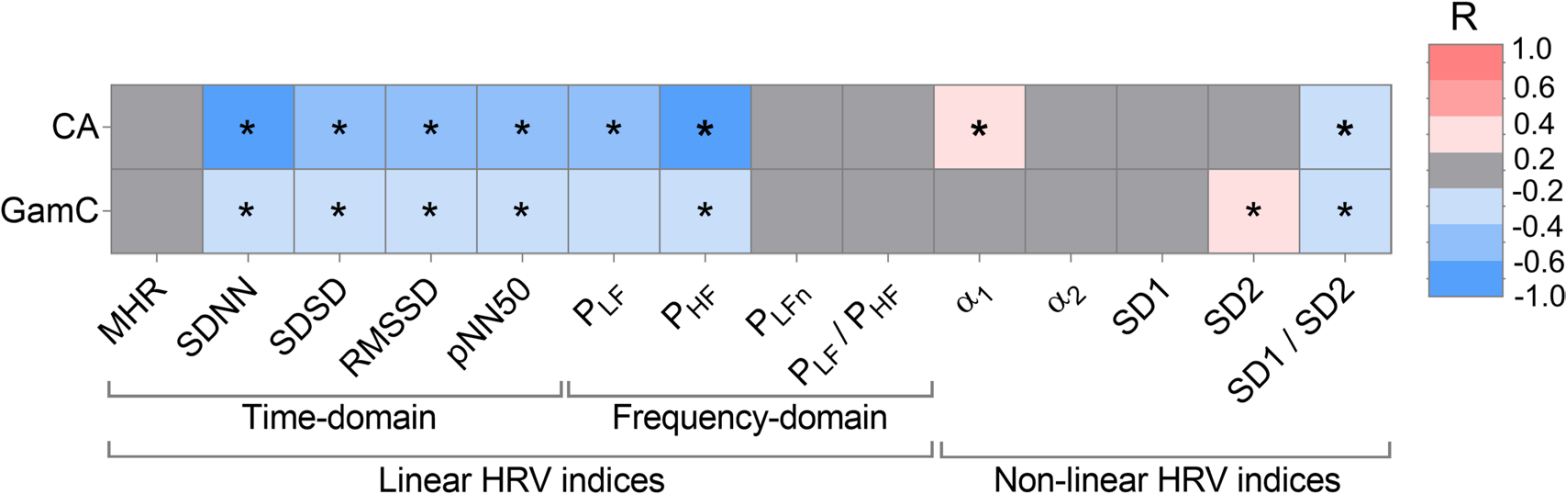
Evaluation of the association of age with HRV parameters. Spearman’s correlation coefficients (*R*) of HRV parameters with CA or GamC are colour coded according to the scale. Significant correlations (FDR≤0.05) are marked by an asterisk. The groups of HRV parameters are delimited below as linear (time domain or frequency domain) and non-linear (MHR: mean heart rate; SDNN: standard deviation of NN intervals; SDSD: standard deviation of the differences between adjacent NN intervals; RMSSD: root mean square of consecutive differences of adjacent NN intervals; pNN50: percentage of consecutive NN intervals differing by more than 50 ms divided by the total number of all NN intervals; *P*_*LF*_: power in the low frequency (LF) band; *P*_*HF*_: power in the high frequency (HF) band; *P*_*LFn*_: normalized *P*_*LF*_ with the total power; *P*_*LF*_/*P*_*HF*_: ratio *P*_*LF*_/*P*_*HF*_; *α* and *α*: short- and long-term correlations between successive RR intervals, respectively; SD1 and SD2: short-term and long-term beat-to-beat variability of the RR interval series, respectively; SD1/SD2: ratio SD1/SD2)

Consistent with this observation and the established hypothesis, CA was only significantly associated with PA-related HRV markers, especially linear temporal and absolute frequency domain markers, including SDNN, SDSD, RMSSD, pNN50, *P*_*LF*_, *P*_*HF*_, and SD1/SD2 (*R* around − 0.6 and FDR ≤ 0.05) (Fig. 4 and Table S10−11). This suggests that CA can moderately explain HRV data in the study cohort, which are influenced by the individuals’ PA levels. GamC largely replicated these findings, but with even weaker power than CA (*R* between − 0.2 and − 0.4) (Fig. 4 and Table S10−11), indicating that GamC marginally described the ANS function.

### GamC is associated with PA levels and modestly responds to a healthy-aging exercise intervention in centenarians

**Fig. 5:**
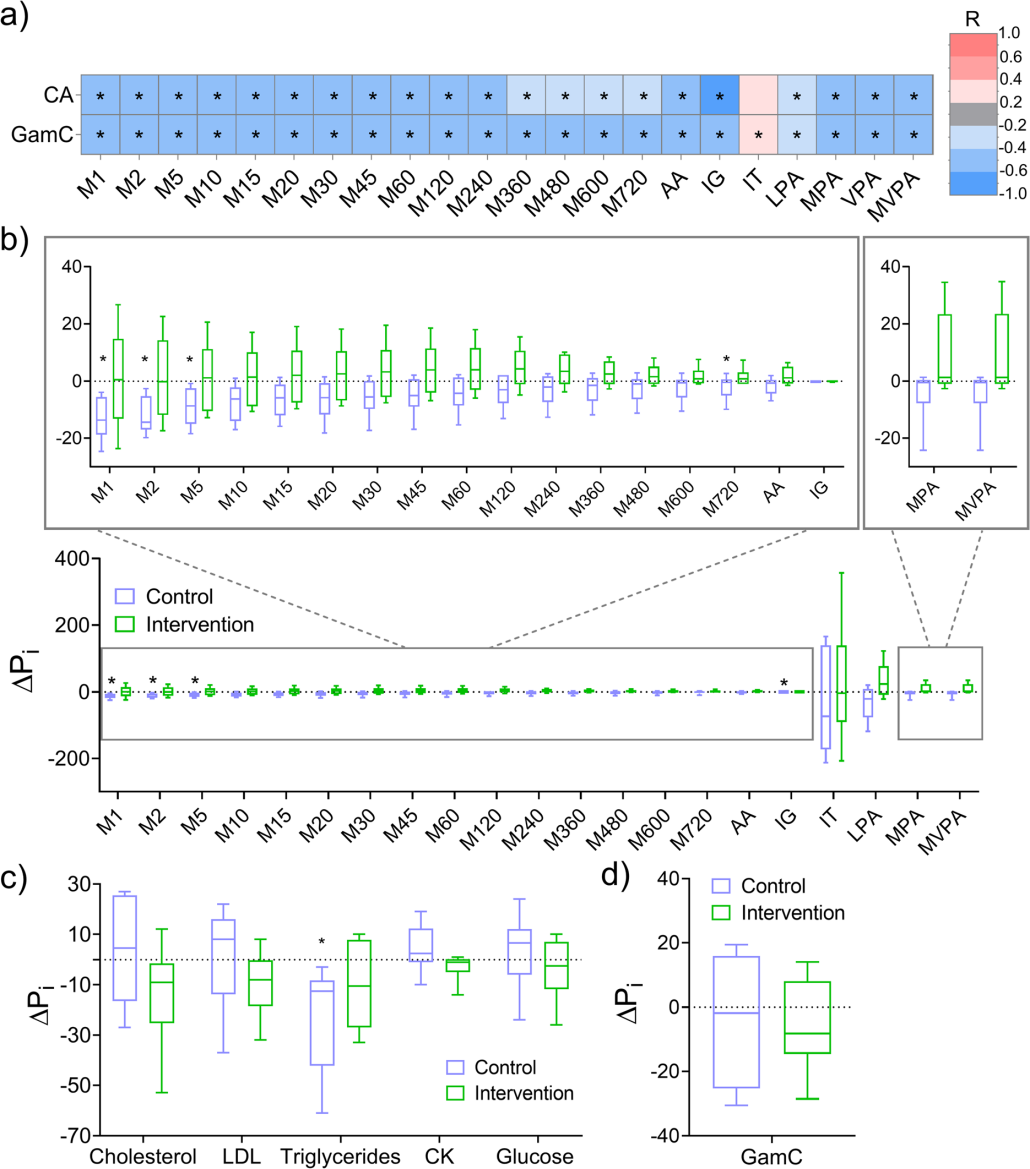
Evaluation of the association of age with parameters of physical activity and healthy aging intervention study in centenarians.**a** Evaluation of the association between accelerometer-derived variables indicative of PA levels with CA and GamC by Spearman correlation analysis. Correlation coefficients (*R*) are colour coded according to the scale and significant independent associations (FDR ≤ 0.05) are marked with an asterisk. **b-d** Effect of the healthy-aging intervention (strength exercise training for 3 months) in centenarians on the b PA levels, c biochemical parameters related to cardiovascular heatlh and d GamC in each control and intervention group. Box plots and whiskers represent the median and 2.5–97.5 percentiles of the differences after (II)-before (I) intervention (*ΔP*_*i*_). Significance results from Wilcoxon paired test (*P* ≤ 0.05) for each control and intervention groups are marked with an asterisk. (M1 to M270: MX metrics from 1 to 720 min; *AA*: average acceleration; *IG*: intensity gradient; *IT*: inactive time; *LPA*: light intensity PA; *MPA*: moderate intensity PA; *VPA*: vigorous intensity PA; *MVPA*: moderate-vigorous intensity PA)

The relationship of GamC with accelerometer variables, as a direct measure of PA levels and of CV health was then analyzed (Fig. 1). CA was significantly correlated with all PA markers (FDR ≤ 0.05 and *R* < − 0.4 for most markers) except for IT (*R* = 0.20). GamC largely replicated this relationship, with an even stronger significant association with IT (*R* = 0.39) (Fig. 5a and Table S12−13). CA and GamC showed comparable sensitivity with respect to short-term metrics (from M1 to M240). However, GamC was more sensitive than CA to long-duration MX metrics (from M360 to M720), demonstrating a better description of the acceleration at longer active periods. AA and IG, as standardized variables to describe the volume and intensity of PA, also showed their moderately negative and significant association with GamC (*R* = − 0.44 and − 0.52, respectively). In terms of PA intensities, GamC showed a weaker correlation with LPA (*R* = − 0.33) than with MPA, VPA, and MVPA (*R* = − 0.47, − 0.46, and − 0.46, respectively), suggesting that GamC may reflect PA of higher intensities with greater precision. This fact is also observed in the associations with CA, which shows stronger associations with MPA, VPA, and MVPA (*R* = − 0.49, − 0.51, and − 0.48, respectively) than with LPA (*R* = − 0.29). Therefore, GamC is able to describe individual PA levels in terms of MX metrics, AA, IG, and IT to the same extent as CA, but with improved sensitivity in some aspects over CA. In addition, the biological nature of GamC is better at describing activities of longer duration and higher intensity than those of shorter duration and lower intensity.

We then examined the potential of GamC to monitor the effect of a healthy aging intervention in centenarians (Fig. 1), since the significance of the findings in this age group is potentially higher than in younger one [54]. Individuals in the centenarian group were randomized to follow (intervention group) or not follow (control group) a 3 months of strength exercise training. The intervention was based on the fact that exercise, including strength training [55], promotes CV health and is associated with a lower risk of all-cause mortality [56], but also that the effects of aging are modifiable [4, 57]. The intervention showed a global beneficial effect in the trained group (Fig. 5b-c and Table S14-15). As expected for the age-related physical decline, the PA variables mostly worsened after the intervention in the control group with a general decrease of the median *ΔP*_*i*_ of PA variables (some of them being significant) with large average effect sizes (|$\overline{r }$|= 0.6) (Fig. 5b and Table S13). However, the intervention group seemed able to buffer and moderately counteract this decline after the exercise-training period. Namely, an opposite trend of the median *ΔP*_*i*_ of the PA variables was observed (general increased values) that was accompanied by medium average effect sizes (|$\overline{r}$|= 0.3) (Fig. 5b and Table S15). In addition, well established biochemical parameters associated with increased risk of CVD, including cholesterol, LDL, CK and glucose, also worsened after the intervention in the control group showing moderate average effect sizes (|$\overline{r }$|=0.4) (Fig. 5c and Table S15), but generally improved in the intervention group. Decreased median *ΔPi* values of cholesterol, LDL, CK and glucose with large average effect sizes (|$\overline{r }$|=0.5) were observed in the intervened group (Fig. 5c and Table S15). Interestingly, the levels of triglycerides decreased in both control and intervention groups, which would need further investigation.

The improvement in the PA metrics and biochemical parameters in the intervention group was translated into an apparent decrease of the GamC *ΔP*_*i*_ median value (median (IQR) = − 8.2 (12.8) years) with a medium effect size (|*r*|= 0.4), that was greater and less dispersed than the differences observed in the control group (median (IQR) = − 1.8(31.3) years) with a small effect size (|*r*|= 0.2) (Fig. 5c and Table S15).

Overall, GamC describes the current PA levels of individuals with some improvements over CA and shows a capacity to respond to an effective healthy aging intervention in centenarians, highlighting its biological relevance.

## Discussion

In our aging societies, the need to promote healthy aging claims for objective biomarkers that monitor the aging process beyond the simple recognition of age as indicated by the date of birth. This need is underpinned by the individual AR, which this work confirms is consistently not associated with CA. Here we report GamC, a simple blood-based BOA that describes the current BA of individuals in terms of CV health-related markers, specifically those that report the PA levels of individuals.

The age-related mechanisms observed in different populations may vary considerably [16, 17]. However, the association between GamC and CA remains consistent across study populations of different geographic origins. This is important because reliable BOAs with potential broad translational application are expected to demonstrate such consistency [58].

The observed association between GamC and CA is not as high as other established age predictors (e.g., PhenoAge and GrimAge [2, 8]). However, a partial correlation with CA is consistent with an important consideration in the development of BOAs. They should be strongly, but not perfectly, related to CA in order to represent the age-related physiological variability of each individual [1]. However, the cut-off point for this distinction is unknown. Our group previously reported AppAge, a cardiac transcriptomic BOA calculated as GamC, but using almost 3000 genes [29]. AppAge had a correlation coefficient value with CA similar to that of GamC in the cohorts used in this study, namely, *R* = 0.47, but described age-related cardiac transcriptomic changes and an aging phenotype. Similar but less sensitive results were observed for a single gene (*CDKN2A)* based BOA (*R* = 0.24 with CA), suggesting that even a single functionally relevant age-related gene can describe molecular changes of cardiac aging. In other words, a high correlation of BOAs with CA does not necessarily imply a better ability to explain the biology of aging. Conversely, a less-than-perfect association may actually leave room for a biologically based personalized description of an individual's aging status. In this regard, GamC, which has correlation coefficients with CA ranging from 0.45 to 0.59 in the studied cohorts, but which describes both changes in biological processes at the transcriptomic level and phenotypes related to the CV health of individuals (specifically PA levels), is calculated based on the expression levels of only three age-related genes (*ABLIM*, *CCR7*, and *LEF1*). These genes are consistently dysregulated in CA in all the independent cohorts considered in this work [11, 45–47, 50–52]. In addition, our data show that they contribute synergistically to the meaning of GamC, as their independent association with CA is improved by their combination in the BOA. GamC, in turn, demonstrates a differential nature, since it does not equally describe all age-related functional data of the study cohort. It describes the PA levels even better than CA for some PA-related variables, but is only marginally sensitive for ANS functional markers. This can most likely be explained by the age-related gene-specific contribution of *ABLIM*, *CCR7*, and *LEF1* to the biological meaning of GamC. Whether GamC can describe other age-related functional data requires further investigation. But indeed, our results support the idea that based on the biological data used to construct BOAs, each BOA is expected to describe different and/or complementary aspects of aging [1]. Taken together, our data suggest that *ABLIM*, *CCR7*, and *LEF1* are functionally relevant age-related genes able to capture different aspects of an individual’s aging biology in GamC, specifically PA-related, and, consequently, GamC is able to describe synchronous functional aspects of individuals regardless of (or perhaps exploiting) its partial correlation with CA. GamC is therefore able to estimate the CV-related BA of individuals in real time in terms of their PA levels.

Although the significance of the outcome of our intervention study in centenarians is limited by the small size of the study groups, it encourages further research into the ability of GamC to monitor BA in response to healthy aging interventions. This is supported by the non-significant decrease of themedian difference of GamC in the intervention group after the intervention, which accompanies the overall beneficial effect of exercise observed in their PA-related variables and biochemical parameters. Therefore, GamC’s sensitivity to exercise training seems remarkable considering that the size of the effect of the intervention on the PA levels is mostly medium too. Whether an intervention with a greater effect size (perhaps a longer training) would translate in greater changes in GamC in centenarians and whether the findings in centenarians hold true for other age groups remains to be explored.

For such purpose and beyond, the simplicity and consequent affordability of GamC (a blood sample and PCR test accessible to any basic molecular laboratory), together with the fact that its biological relevance is determined in relation to synchronous functional data rather than with mortality risk or disease outcome, makes GamC a valuable translational tool with potential application in real-time clinical decision-making and intervention monitoring. GamC is therefore a relevant candidate BOA for promoting healthy aging in relation to CV health, the leading cause of death worldwide.

## Limitations of the study

We show that *ABLIM*, *CCR7*, and *LEF1* expression is related to age in a gender-independent manner; however, the low presence of females in the study cohort may limit our conclusions.

The small number of centenarians in the study limits the conclusions of the healthy-aging intervention, which was performed only in this age group.

GamC shows BOA potential in the study in relation to PA levels, but this needs to be tested in independent and larger populations.

## Conclusion

We develop and characterize a simple and cost-effective BOA based on gene expression in blood, GamC, which meets capabilities of an aging clock. Specifically, it is able to describe CA on a biological (molecular) basis in independent cohorts; it associates with age-related functional aspects relevant to the current CV health status of individuals (PA levels) and shows moderate responsiveness to a healthy aging intervention. In conclusion, we propose GamC as a suitable candidate BOA for future research to determine the CV health risk in relation to BA in real time, as well as to monitor the effect of healthy aging interventions, which is beyond the capabilities of CA. Its simplicity enhances its clinical translational value to large populations.

## Supplementary Information

Below is the link to the electronic supplementary material.

Supplementary file1 (DOCX 580 KB)

Supplementary file2 (XLSX 451 KB)

## Data Availability

Data availability statement is not included, as all data is included in the supplementary files.
