# Supplementary material for: A simple blood biomarker based on gene expression describes cardiovascular health-related biological age
Source: GeroScience. 2025 Jul 29;47(5):6613–29. doi: 10.1007/s11357-025-01784-6 (PMC12635007; doi:10.1007/s11357-025-01784-6)
Supplement: Supplementary file 1 — Supplementary file1 (DOCX 580 KB) [file 11357_2025_1784_MOESM1_ESM.docx]

***Supplementary material***

**
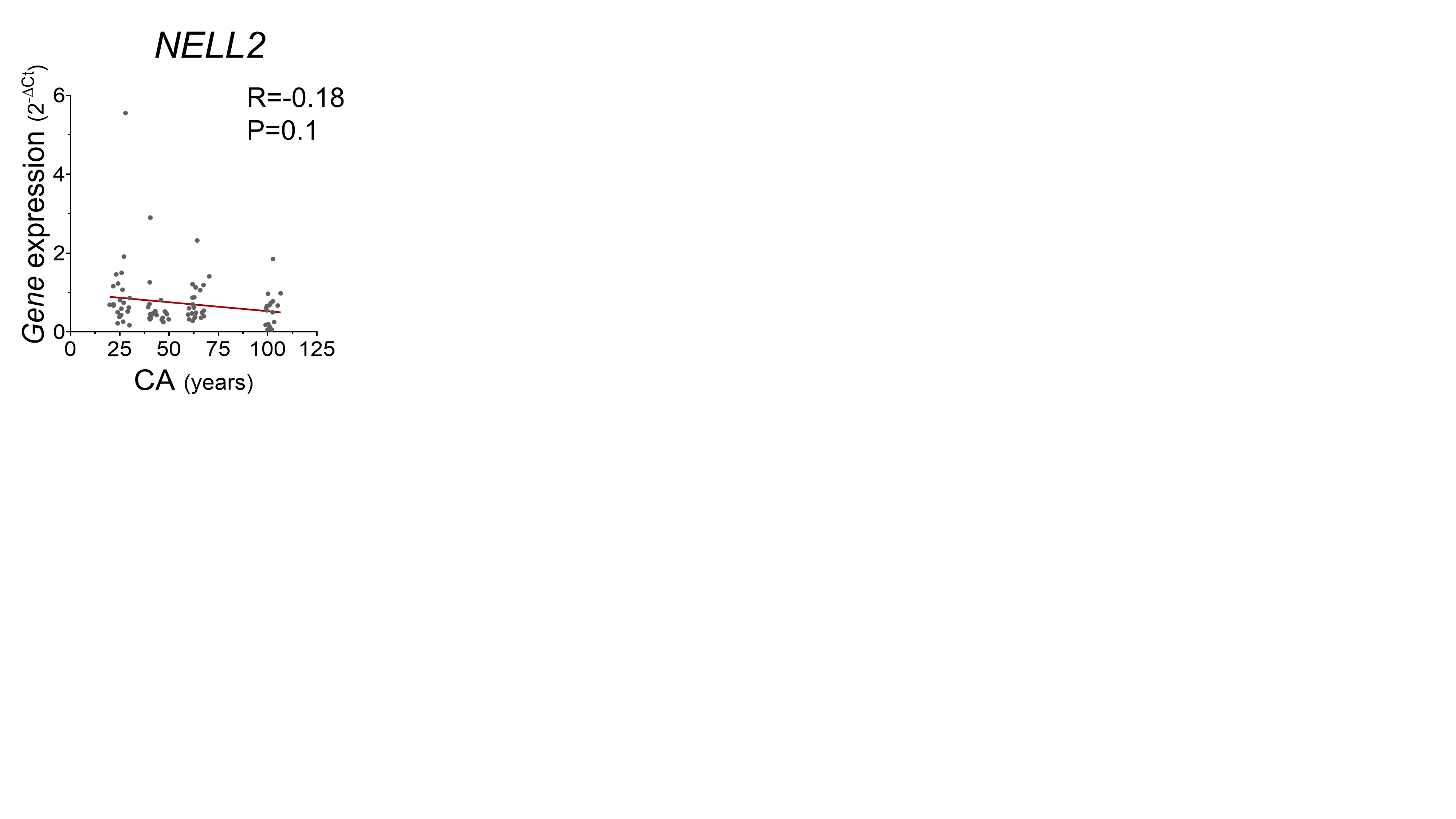
**

**Fig S1 Expression levels of *NELL2* and association analysis with CA in the study cohort.** Pearson´s correlation coefficient (R) and p-value (P) are shown. Linear regression is represented as a red line.

**
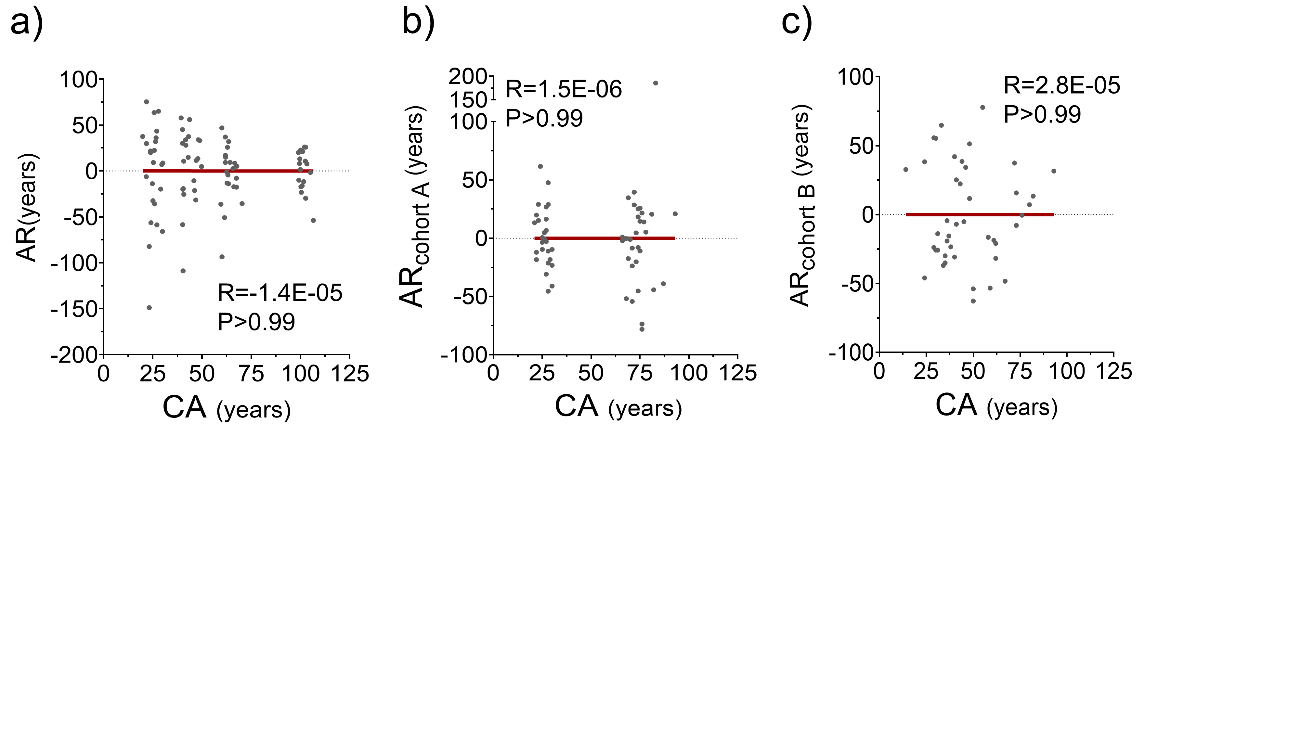
**

**Fig S2** **Association of the aging rate with CA.** **a)** Pearson correlation analysis of the aging rate with CA in a) the study cohort and validation cohorts **b)** A and **c)** B. The Pearson´s correlation coefficient (R), p-value (P) and linear regression (red line) are shown.

**
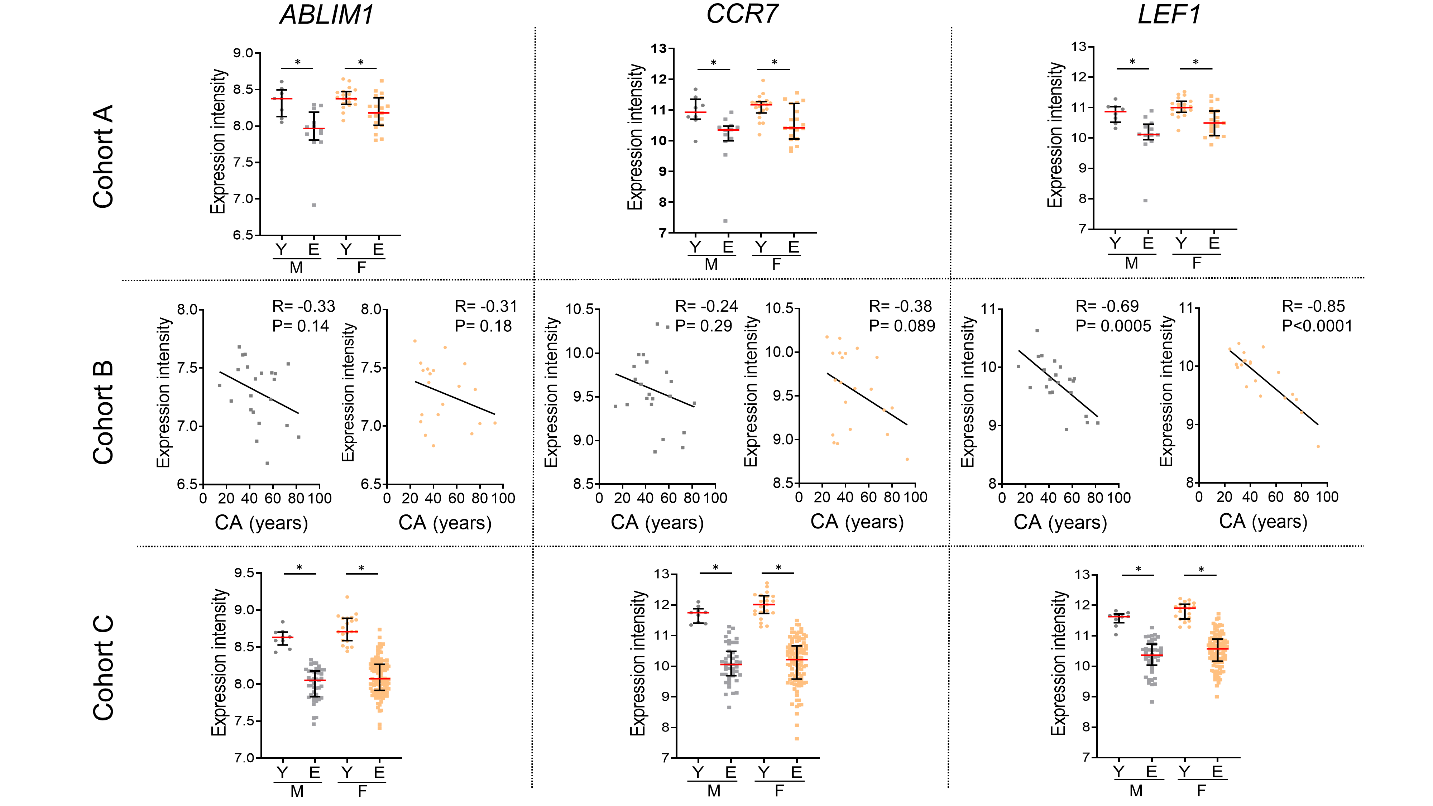
**

**Fig S3** **Normalized and standardized expression values of *ABLIM1*, *CCR7* and *LEF1* in blood samples of the individuals of the three independent validation cohorts.** Cohorts A and C contain young (Y) and elder (E) age groups, while cohort B is constituted with individuals of continuous ages. Genders are colour coded: males (M) in grey and females (F) in orange. Pearson correlation analysis was done for cohort B and the correlation coefficients (R), p-values (P) and linear regressions (red line) are shown for each comparison. Mann-Whitney U test was used for cohorts A and C and significant differences between groups (P ≤ 0.05) are marked by an asterisk.

**
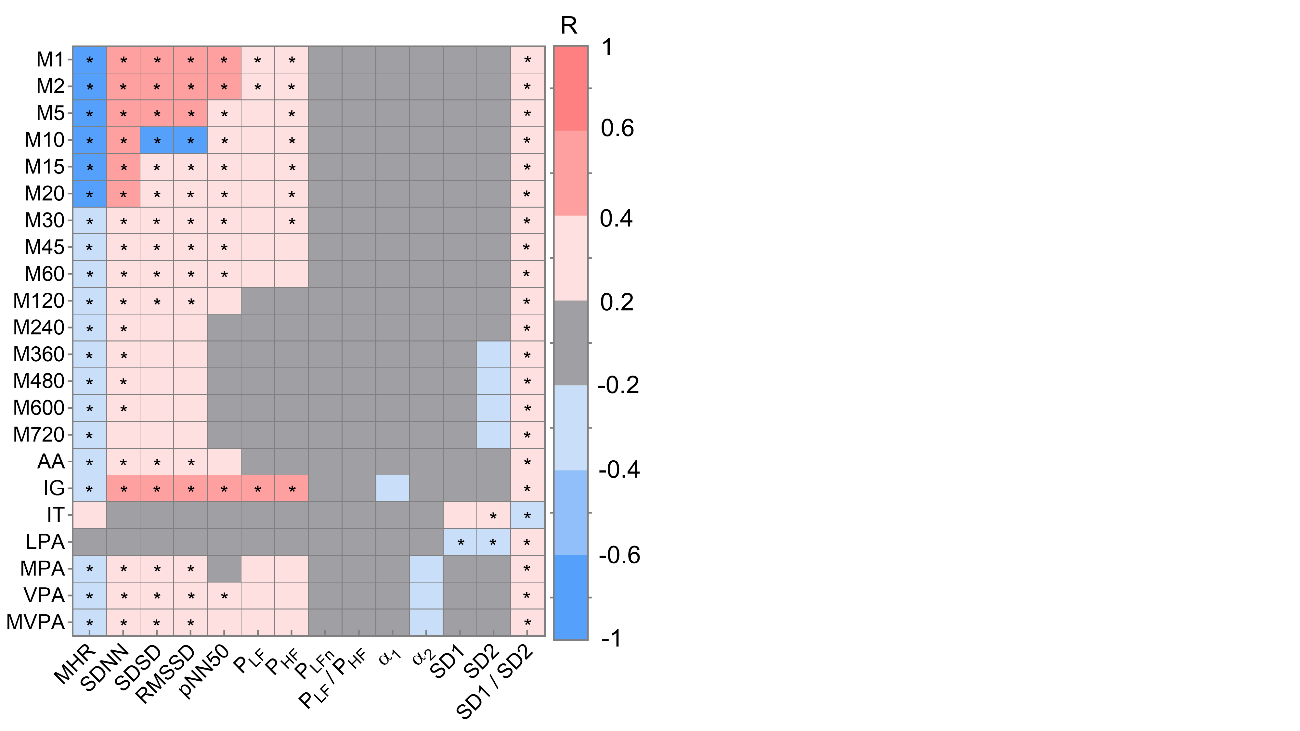
**

**Fig S4 Independent association analysis between accelerometer metrics and HRV parameters in the study cohort.** Spearman correlation coefficients (R) are colour coded according to the scale and significant (FDR≤0.05) relationships are marked with an asterisk. (MHR, mean heart rate; SDNN, standard deviation of NN intervals; SDSD, standard deviation of the differences between adjacent NN intervals; RMSSD, root mean square of consecutive differences of adjacent NN intervals; pNN50, percentage of consecutive NN intervals differing by more than 50 milliseconds divided by the total number of all NN intervals; P_LF_, power in the low frequency (LF) band; P_HF_, power in the high frequency (HF) band; P_LFn_, normalized P_LF_ with the total power; P_LF_/P_HF_, ratio P_LF_/P_HF_; α_1_ and α_2_, short- and long-term correlations between successive RR intervals, respectively; SD1 and SD2, short-term and long-term beat-to-beat variability of the RR interval series, respectively; SD1/SD2, ratio SD1/SD2).

**Table S1 Data of the study cohort used in the GamC calculation and characterization sections of the workflow.** Demographic data, anthropometric measures, gene expression data and age biomarkers in the study cohort (GamC and AR).

**Table S2 List of oligonucleotides used as primers for qPCR.**

**Table S3** Relationship between the nomenclature used in this paper and the variable names from the GGIR output.

**Table S4 Description of the validation cohorts A, B and C.** Demographic data of the cohorts (blue background): study origin, age groups, age range, number of individuals (% of males)). Analysis of the relationship of *ABLIM*, *CCR7* and *LEF1* expression levels with CA and gender (grey background): decreased expression in elderly compared to young individuals is represented as ↓ followed by the p-value of the Mann-Whitney U test (cohorts A and C). Pearson correlation coefficients with CA/p-value is shown for cohort B. Age groups used in the differential expression analysis (DEA) and gene set enrichment analysis (GSEA) of each cohort for both CA and GamC (green background): age and number of individuals (% of males).

**Table S5 Studies reporting age-related changes in the blood transcriptome found in the identification phase of the workflow.** List of published studies used for the identification of candidate age-related genes: sample type, dysregulated genes common across studies in this list, geographical origin, age group, age range, number of individuals (% of males) and reported CA-dysregulated processes.

**Table S6 Data of individuals from cohorts A, B and C**. GEO accession, CA, race, country, gender, normalized and standardized expression values of *ABLIM1*, *CCR7* and *LEF1* and values of GamC and the AR.

**Table S7 GSEA results from cohorts A and B ranked by CA and GamC.** NES, normalized enrichment score; P, p-value; FDR, false discovery rate; Q, q-value.

**Table S8** GO classification in general functional categories in cohort A and B.

**Table S9 Association analysis between accelerometer data and HRV indices.** R, Spearman correlation coefficients; FDR, false discovery rate.

**Table S10 Raw HRV data from the study cohort.** Parameters are classified in linear (time- and frequency domain parameters) and non-linear domain measures (min, minutes, ms, milliseconds).

**Table S11 Association analysis between HRV markers and CA or GamC in the study cohort**. R, Spearman coefficient correlations; FDR, false discovery rate.

**Table S12 Raw accelerometer data from the study cohort.**

**Table S13 Association analysis between accelerometer data and CA or GamC in the study cohort**. R, Spearman correlation coefficients; FDR, false discovery rate.

**Table S14 Raw accelerometer and biochemical data from the centenarians participating in the intervention study.** The values before (I) and after (II) the intervention period (three months) are indicated for each individual in both control (not undergoing the intervention) and intervention (undergoing the strength training exercise) groups.

**Table S15** **Evaluation of the effect of the intervention in centenarians in terms of levels of physical activity (PA), biochemical parameters (BQ) and values of GamC.** The table indicates: Median (IQR) of the change in each parameter after the intervention period (ΔP_i_) (P, p-value of the Wilcoxon matched-pairs signed rank test; |r|, effect size estimated by the Wilcoxon matched-pairs signed rank test; | r̅ |, average of effect size).
